# miR-212 and miR-132 Are Downregulated in Neurally Derived Plasma Exosomes of Alzheimer’s Patients

**DOI:** 10.3389/fnins.2019.01208

**Published:** 2019-11-26

**Authors:** Diana J. Cha, David Mengel, Maja Mustapic, Wen Liu, Dennis J. Selkoe, Dimitrios Kapogiannis, Douglas Galasko, Robert A. Rissman, David A. Bennett, Dominic M. Walsh

**Affiliations:** ^1^Laboratory for Neurodegenerative Disease Research, Ann Romney Center for Neurologic Diseases, Brigham and Women’s Hospital, Harvard Medical School, Boston, MA, United States; ^2^Department of Neurodegenerative Diseases, Hertie-Institute for Clinical Brain Research and Center of Neurology, University of Tübingen, Tübingen, Germany; ^3^German Center for Neurodegenerative Diseases, University of Tübingen, Tübingen, Germany; ^4^Laboratory of Neurosciences, National Institute on Aging, National Institutes of Health, Baltimore, MD, United States; ^5^Department of Neurosciences, University of California, San Diego, La Jolla, CA, United States; ^6^VA San Diego Healthcare System, La Jolla, CA, United States; ^7^Rush Alzheimer‘s Disease Center, Rush Medical College, Chicago, IL, United States; ^8^Alzheimer’s Disease and Dementia Research Unit, Biogen Inc., Cambridge, MA, United States

**Keywords:** blood biomarker, extracellular vesicles, L1CAM, micro-RNA, mild cognitive impairment, qRT-PCR

## Abstract

It was recently discovered that brain cells release extracellular vesicles (EV) which can pass from brain into blood. These findings raise the possibility that brain-derived EV’s present in blood can be used to monitor disease processes occurring in the cerebrum. Since the levels of certain micro-RNAs (miRNAs) have been reported to be altered in Alzheimer’s disease (AD) brain, we sought to assess miRNA dysregulation in AD brain tissue and to determine if these changes were reflected in neural EVs isolated from blood of subjects with AD. To this end, we employed high-content miRNA arrays to search for differences in miRNAs in RNA pools from brain tissue of AD (*n* = 5), high pathological control (HPC) (*n* = 5), or cognitively intact pathology-free controls (*n* = 5). Twelve miRNAs were altered by >1.5-fold in AD compared to controls, and six of these were also changed compared to HPCs. Analysis of hits in brain extracts from 11 AD, 7 HPCs and 9 controls revealed a similar fold difference in these six miRNAs, with three showing statistically significant group differences and one with a strong trend toward group differences. Thereafter, we focused on the four miRNAs that showed group differences and measured their content in neurally derived blood EVs isolated from 63 subjects: 16 patients with early stage dementia and a CSF Aβ42+ tau profile consistent with AD, 16 individuals with mild cognitive impairment (MCI) and an AD CSF profile, and 31 cognitively intact controls with normal CSF Aβ42+ tau levels. ROC analysis indicated that measurement of miR-132-3p in neurally-derived plasma EVs showed good sensitivity and specificity to diagnose AD, but did not effectively separate individuals with AD-MCI from controls. Moreover, when we measured the levels of a related miRNA, miR-212, we found that this miRNA was also decreased in neural EVs from AD patients compared to controls. Our results suggest that measurement of miR-132 and miR-212 in neural EVs should be further investigated as a diagnostic aid for AD and as a potential theragnostic.

## Introduction

Alzheimer’s disease is a devastating disorder for which there is no cure or effective treatment. Symptom onset is insidious and even in sophisticated centers clinical diagnosis is imperfect (Salloway et al., 2014; Monsell et al., 2015). Advances in brain imaging and the development of robust immunoassays to measure tau and amyloid β-protein (Aβ) in cerebrospinal fluid (CSF) have greatly aided diagnosis (Blennow et al., 2012). Specifically, measurement of tau and Aβ in CSF, or quantitation of amyloid or tangle pathology by PET imaging, can be used to identify mild cognitive impairment (MCI), a frequent precursor of AD (Jack et al., 2013; Bao et al., 2017), and use of these markers is now common in clinical research (Blennow et al., 2012; Jack et al., 2013). But PET imaging is expensive and is restricted to use in certain geographies. Assessment of markers in CSF is more amendable for general use, but CSF sampling remains unpopular with patients. Thus, there is a pressing need for less costly and intrusive, and more widely available biomarkers that can replace or supplement current CSF and PET markers (Bateman et al., 2019). Measurement of a blood-based analyte would be ideal since blood collection is widely accepted by patients and can be done almost anywhere.

Unlike CSF, the contents of blood are influenced by many organs and therefore changes in blood analytes are often not sensitive to minor changes that occur in brain. For instance, while the levels of Aβ42 in CSF change early in AD, measurement of Aβ42 in plasma has not proved as useful (Janelidze et al., 2016). The discovery that brain cells release extracellular vesicles (EVs) and a portion of these enter the bloodstream offers the potential of monitoring changes occurring in the brain by isolating EVs from venous blood. EVs are small packages of cytosol encapsulated by a lipid bilayer and are released by all cells, including neurons and glia (Fruhbeis et al., 2012; Raposo and Stoorvogel, 2013; Paolicelli et al., 2019). *Exosome* is the term most frequently applied to EVs, but there are many classes of EVs, primarily defined by their cellular origin. Exosomes arise through the endolysosomal pathway and are specifically formed by inward budding of multivesicular bodies to produce intraluminal vesicles. After maturation, multivesicular bodies can fuse with the plasma membrane and exosomes are released (Pan et al., 1985; Johnstone et al., 1987).

A series of recent papers on neural exosomes isolated from blood plasma (Fiandaca et al., 2015; Goetzl et al., 2015a, b; Kapogiannis et al., 2015; Abner et al., 2016) has caused huge excitement among the neuroscience biomarker community (Galasko, 2015; Heinzelman et al., 2016; Vella et al., 2016). The method used employs a proprietary reagent, ExoQuick, to precipitate total “exosomes” from plasma, followed by enrichment of material of “neural” origin using an antibody to the neural cell adhesion molecules, L1CAM (Fiandaca et al., 2015). In two independent studies, analysis of samples prepared using this protocol revealed significant increases in pT181-tau, pS396-tau and Aβ1-42 in patients with AD vs. those from age-matched normal cognitively intact controls (Fiandaca et al., 2015; Winston et al., 2016). Other protein cargo in neural exosomes have also been found to change in AD (Goetzl et al., 2019). However, the ability to discriminate AD from AD-MCI and controls required a statistical approach that constrains comparison across cohorts analyzed on different occasions. Furthermore, a more recent publication employing a similar method failed to detect elevation of pT181-tau or Aβ1-42 in neural exosomes from AD subjects (Guix et al., 2018).

Micro-RNAs (miRNA) are small non-coding RNA molecules which base-pair with complementary sequences within mRNA to decrease post-transcriptional gene expression (O’Brien et al., 2018) and accumulating evidence indicates that the expression of certain miRNAs are altered in AD (Lau et al., 2013; Patrick et al., 2017; Pichler et al., 2017; El Fatimy et al., 2018; Swarbrick et al., 2019). However, it is unknown whether miRNA dysregulation in the brain is reflected in neural exosomes. To this end, we performed an unbiased analysis of miRNAs in human brain tissue to inform miRNA candidates for targeted analysis in neurally derived plasma exosomes. First, we measured miRNAs in pooled RNA isolated from five individuals with AD, five cognitively intact high pathology controls (HPCs), and five pathology-free controls. HPC refers to subjects with sufficient amyloid plaques and neurofibrillary tangles to be diagnosed as AD, but at the time of death these individuals had no evidence of cognitive impairment. Recently, it had been suggested to consider such individuals as AD stage 1 and 2 (Jack et al., 2018). Results were highly similar across the pooled and individual brain samples. In a total of 27 individual brain samples: 9 controls, 7 HPC and 11 AD, miR-182-5p, miR-591, miR-32-3p, and miR-132-3p were dysregulated in AD vs. both controls and HPC.

Armed with this information, we asked whether the miRNAs changed in AD brain were also altered in neural exosomes isolated from plasma. Since, diagnosis of AD based on clinical criteria alone is uncertain (Monsell et al., 2015) we were careful to use specimens from individuals whose clinical diagnosis was confirmed by the best current biomarkers – CSF Aβ42 and tau. Using plasma from patients that conformed to these criteria we prepared neural exosomes from 31 cognitively intact biomarker-negative controls, 16 CSF biomarker-positive MCI, and 16 CSF biomarker-positive AD. RNA was extracted from neural exosomes, reversed transcribed and analyzed by qRT-PCR for the four miRNAs most altered in AD brains. miR-132 was decreased by ∼9-fold in AD exosomes vs. controls, and ∼5.4 fold in AD vs. AD-MCI. Since miRNAs within the same cluster are often co-regulated, we also measured miR-212, a miRNA in tandem to miR-132. Strikingly, the levels of miR-212 were reduced fourfold in AD samples compared to controls. These results suggest that concerted dysregulation of miR-132 and miR-212 occurs in AD and that measuring the levels of these miRNAs in neurally derived exosomes may offer a window on changes occurring in AD brain. Our results in neural exosomes are consistent with prior studies that detected down-regulation of miR-132 and miR-212 in AD brain (Pichler et al., 2017) and down-regulation of miR-132 in blood (Denk et al., 2018).

## Materials and Methods

### Brain Donors

Human specimens were obtained from Rush Alzheimer’s Disease Center, Rush University Medical Center and used in accordance with the Partners Institutional Review Board (Protocol: Walsh BWH 2011). Samples of frozen frontal cortex were from the Religious Orders Study (ROS), a longitudinal clinical-pathological cohort study in aging and Alzheimer’s Disease (Bennett et al., 2018). Participants from religious communities were enrolled at a time when they were free of known dementia. The study was approved by the Institutional Review Board of Rush University Center. All participants signed an informed consent, Anatomic Gift Act, and a repository consent to allow their data and biospecimens to be shared for research. They were followed over time until brain donation. Overall follow-up and autopsy rates were about 95%. Each participant underwent uniform structured annual cognitive and clinical evaluations. A diagnosis AD dementia required evidence of meaningful cognitive decline in two domains of cognition, one of which was episodic memory. Clinical summary diagnostic opinion was made *post-mortem* based on all available clinical information by a neurologist blinded to pathologic data. A neuropathologic diagnosis of high (1), intermediate (2), low (3), or no AD (4) was based on the modified NIA-Reagan Diagnosis of AD. This assessment relies on the severity and distribution of both neurofibrillary tangles and neuritic plaques (Bennett et al., 2006). Individuals were categorized as controls, high pathology controls (HPC), or AD based on combined clinical and pathological criteria (Table 1). Individuals with an NIA-Reagan score of 1 or 2 and a clinical diagnosis of AD dementia were defined AD (*n* = 11), whereas patients with an NIA-Reagan Score of 1 or 2 and no evidence of dementia were designated HPC (*n* = 8). Patients in the control group had an NIA-Reagan score of 3 and no evidence of dementia.

**TABLE 1:** Demographics and clinical characteristics of brain donors.

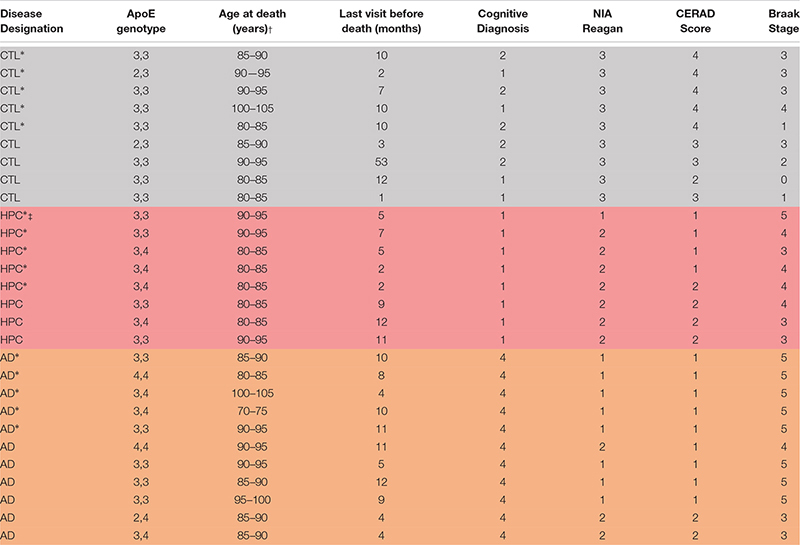

*CTL, control; HPC, high pathology control; AD, Alzheimer’s Disease; Cognitive Diagnosis: 1 (no cognitive impairment), 2 (mild cognitive impairment), 4 (Alzheimer’s Disease Dementia); NIA-Reagan: 1 (high likelihood of AD), 2 (intermediate likelihood of AD), 3 (low likelihood of AD); CERAD score: 1 (definite AD), 2 (probable AD), 3 (possible AD), 4 (no AD); Braak stage: 0 (no NFT pathology), I and II (NFT pathology confined to the enthorinal region), III and IV (involvement of limbic regions), V and VI (moderate to severe neocortical involvement). ^∗^Used for miRNA high content arrays †age-range is given to preserve anonymity of participants. ‡This brain was used only for the pooled analysis and not for assessment of individual brains. Gray represents brain samples from the control group, pink represents HPC, and orange represents AD.*

### Study Participants, CSF and Blood Collection

Specimens were from research participants in the UCSD Shiley-Marcos Alzheimer’s Disease Research Center, collected and used in accordance with IRB approval (Table 2). Each participant donated both ETDA plasma and CSF, which were obtained using standardized protocols. CSF was collected into polypropylene tubes, centrifuged at 1,500 × *g* for 10 min, and aliquoted into polypropylene storage tubes. Blood was drawn on the same day as lumbar puncture, and plasma was isolated and aliquoted. On the day of analysis, samples were thawed at room temperature, and used for subsequent exosome isolation.

**TABLE 2:** Demographics and clinical characteristics of plasma donors.

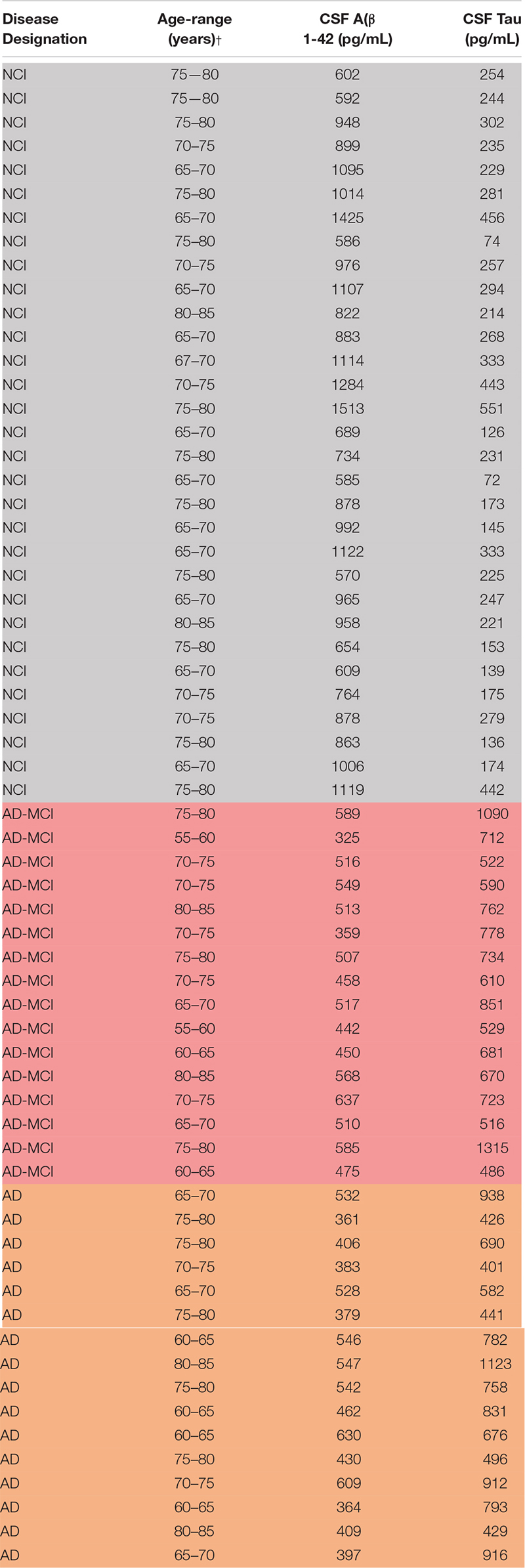

*NCI, no cognitive impairment; AD-MCI, mild cognitive impairment due to Alzheimer’s Disease; AD, Alzheimer’s Disease. †Age-range is given to preserve anonymity of participants.*

Subjects were designated as AD, MCI, and controls based on cognitive testing (MMSE) and CSF biomarker criteria. Control subjects (*n* = 16) had an MMSE ≥ 28, a CSF Innotest tau/Aβ ratio < 0.5 and Aβ > 500 pg/mL (mean Innotest tau level: 248.6 ± 111.5 pg/mL; mean Innotest Aβ1-42 level: 911.2 ± 245.1 pg/mL). AD-MCI and AD subjects had a CSF Innotest tau/Aβ ratio ≥ 1 and Aβ < 650 pg/mL. AD subjects (*n* = 31) had a CSF AD biomarker profile (mean Innotest tau level: 699.6 ± 219.7 pg/mL; mean Innotest Aβ1-42 level: 500.0 ± 81.9 pg/mL) and an MMSE score of 15–24 points, whereas AD-MCI (*n* = 16) subjects had a CSF AD biomarker profile (mean Innotest tau level: 723.1 ± 218.8 pg/mL; mean Innotest Aβ1–42 level: 470.3 ± 90.5 pg/mL) and an MMSE score of 25–29 points. CSF levels of Aβ and tau yielded clear separation of diagnostic groups (Supplementary Figure S1).

### Generation of Induced Neurons (iNs) From Human Induced Pluripotent Stem Cells (iPSCs) and Isolation of Exosomes From Conditioned Media

Neurogenin 2 (Ngn2)-induced human neurons were prepared as before (Guix et al., 2018). In brief, YZ1 iPSCs were maintained in medium containing DMEM/F12, Knockout Serum Replacement, penicillin/streptomycin, L-glutamine, MEM-NEAA, and β-mercaptoethanol (all from Invitrogen, Carlsbad, CA, United States) which was supplemented with 10 μg/mL basic fibroblast growth factor (bFGF; Millipore-Sigma, Burlington, MA, United States). For viral infection, iPSCs were plated at a density of 95,000 cells/cm^2^. Ultrapure lentiviral titers (Alstem, Richmond, CA, United States) were used at the following concentrations: Tet-*O*-Ngn2-puro: 0.1 μL/50,000 cells; Tet-*O*-FUW-EGFP: 0.05 μL/50,000 cells; FUdeltaGW-rtTA: 0.11 μL/50,000 cells. Ngn-2 expression was induced by addition of doxycycline on iN day 1 at a concentration of 2 μg/mL. On iN day 2, puromycin was added at 20 μg/mL, and this concentration was maintained thereafter. On iN day 4, cells were plated on Matrigel (Corning, NY, United States)-coated 100 mm dishes (650,000 cells) and cultured in Neurobasal media (Gibco, Carlsbad, CA, United States) containing Glutamax, 20% dextrose, MEM-NEAA, B27 and 10 ng/mL BDNF, CNTF, GDNF (PeproTech, Rocky Hill, NY, United States). Conditioned media (CM) was collected on iN D21-33, pooled, and processed in batches of 30 mL. First, CM was centrifuged at 300 × *g* and 4°C for 10 min to pellet floating cells. Next, the upper 97% was recovered, and centrifuged at 2,000 × *g* and 4°C to pellet apoptotic bodies. Then, the upper 97% was recovered, diluted in neurobasal media, and centrifuged at 10,000 × g and 4°C for 30 min to pellet microvesicles and cell debris. Finally, the upper 97% was recovered and subjected to centrifugation at 100,000 × *g* and 4°C for 70 min to pellet exosomes.

### RNA Isolation and miRNA Analysis From Brain Tissue

Gray matter from frozen cortex was dissected on dry ice. Total RNA was extracted from ∼20 mg gray matter using a miRNeasy Advanced Kit (Qiagen, Louisville, KY, United States) according to the manufacturer’s guidance. Briefly, samples were suspended in 200 μL ice cold RIPA buffer followed by addition of 120 μL kit-provided RPL buffer. Tissue was homogenized by gently pipetting samples up and down. Forty μL kit-provided RPP buffer was added to samples and vortexed for ∼30 s to facilitate precipitation of proteins. Samples were then centrifuged at 12,000 × *g* for 3 min to pellet the precipitate and debris, and approximately 230 (μL of supernatant was transferred to a new 1.5 mL tube. An equal volume of isopropanol was added and mixed by pipetting before transferring the entire sample to an RNeasy UCP MinElute column. Columns containing sample were centrifuged at 12,000 (x g for 1 minute. Flow-through was discarded and columns were washed according to the manufacturer‘’s protocol. RNA was eluted with two rounds of 20 (L RNase-free water. RNA yield and quality were quantified on a nanodrop. Fifty ng/μL aliquots of RNA were prepared and stored at −20°C.

One hundred and fifty ng of RNA was used for reverse transcription (RT) using a miSCRIPT RT II kit (Qiagen, Louisville, KY, United States), where samples were incubated at 37°C for 60 min followed by inactivation at 95°C for 5 min. RT efficiency was determined by measuring levels of miRTC, a RT control.

### RNA Isolation and miRNA Analysis From iN Cells and iN-Derived Exosomes

iN cells were harvested and centrifuged at 300 × *g* for 10 min to pellet cells. Cell pellets were washed with PBS and lysed with RPL buffer. Exosomes from iN CM were isolated by differential centrifugation as described above, washed with PBS, and lysed in RPL buffer. Subsequent steps were followed identically to the isolation of miRNA from brain tissue.

### Isolation of Exosomes and Enrichment of Neuronal Exosomes

To verify that our methods could reliably detect miRNA expression in an exosomal fraction of human plasma, and to optimize preparative and analytical methods prior to processing precious clinical samples, we first tested experimental conditions using human plasma (each in technical duplicate) from two healthy volunteers.

The method to isolate neuron-derived exosomes from plasma has been validated and described in detail before (Guix et al., 2018), and is a slightly modified version of the procedure pioneered by the Goetzl/Kapogiannis group (Kapogiannis et al., 2015). Five μL thrombin (System Bioscience, Palo Alto, CA, United States) was added to 500 μL aliquots of plasma to induce clot formation and allow the removal of fibrin and related proteins. Reactions were mixed by inversion and incubated for 30 min at room temperature before dilution with 495 μL Ca^2+^- and Mg^2+^-free Dulbecco’s Phosphate-Buffered Saline (DPBS) (Sigma-Aldrich, St. Louis, MO, United States) containing 3x phosphatase (Thermo Fisher, Carlsbad, CA, United States) and protease (Roche, Branchburg, NJ, United States) inhibitors. Thereafter, the samples were centrifuged at 6000 × *g* for 20 min at 4°C and the supernatant transferred to a new tube. Next, 252 μL ExoQuick (System Bioscience, Palo Alto, CA, United States) was added to the supernatants, samples mixed by inversion and left to stand at 4°C for 1 h. Vesicles present in the serum were recovered by centrifugation at 1500 × *g* for 20 min at 4°C and resuspended by vortexing in 500 μL MilliQ water containing 3 × phosphatase and protease inhibitors. ExoQuick pellets were then mixed overnight at 4°C on a vertical rotating mixer.

To isolate neuronal exosomes from the suspensions of total plasma exosomes, 4 μg biotinylated anti-L1CAM antibody (eBioscience, San Diego, CA, United States) in 42 μL 3% bovine serum albumin in DPBS (BSA/DPBS) was added to the resuspended vesicles and incubated on a rotating mixer for 1 h at 4°C. Thereafter, 15 μL of pre-washed Streptavidin-Plus UltraLink Resin (Thermo Fisher, Danvers, MA, United States) in 25 μL 3% BSA/DPBS was added to each sample and incubated for 4 h at 4°C with rotation. Neuronal exosomes bound to the antibody/resin complex were recovered by centrifugation at 200 × *g* for 10 min at 4°C, and washed once with 3% BSA/DPBS before elution in 200 μL 0.1 M glycine (pH 3.0). Resin was removed by centrifugation at 4500 × *g* for 5 min at 4°C. Thereafter, 195 μL supernatant was neutralized with 15 μL of 1 M TrisHCl (pH 8.0) and exosomes were lysed by adding 360 μL M-PER (Thermo Fisher, Carlsbad, CA, United States), 25 μL 3% BSA/DPBS containing 1× phosphatase and protease inhibitors, and 2 freeze-thaw cycles before downstream analysis.

RNA isolation from neural exosomes was performed identically to brain samples, with the following modifications: total exosomes (Exoquick pellets), neural exosomes (L1CAM immunoisolates), or irrelevant/non-specific exosomes (46-4 immunoisolates) were mixed with 240 μL RPL buffer, followed by 80 μL RPP buffer. All subsequent steps were performed as for RNA isolation from brain tissue.

Bioanalyzer analysis showed that samples were enriched with small RNA species following elution from RNeasy UCP MinElute columns.

We cannot completely rule out that some plasma RNA outside of exosomes might be isolated along with exosomal RNA in our protocol. However, this seems unlikely, since free-floating RNA is rapidly degraded in plasma, and RNA protected in RNA-binding proteins or lipids would not allow isolation by L1CAM immunoprecipitation.

### miRNA Quantitative PCR Array

The miRNA expression profile of RNA extracted from brain tissue was analyzed using a miScript miRNA PCR Array Human miRBase Profiler HC Plate 1, MIHS-3401Z (Qiagen, Hilden, Germany). Each array contains 372 unique miRNA assays, 3 snoRNA and 3 snRNA housekeeping genes, duplicate RT primer assays to evaluate RT efficiency, and duplicate PCR primer assays to evaluate the efficiency of the qPCR reaction (for a full list of miRNAs detectable using miScript arrays see: https://b2b.qiagen.com/~/media/genetable/mi/hs/34/mihs-3401z). Six μL RNA (initial concentration = 50 ng/μL) was prepared in 20 μL reverse-transcription reactions (final concentration = 15 ng/μL) using miScript HiSpec Buffer where mature miRNAs are polyadenylated by Poly(A)polymerase and reverse transcribed into cDNA using oligo-dT primers. The oligo-dT primers have a 3′ degenerate anchor and a universal tag sequence on the 5′ end, allowing amplification of mature miRNA in real-time PCR. Each reverse-transcription reaction was further diluted with 90 μL RNAse-free water (4.5 fold dilution) before being used as template for real-time PCR analysis on the miScript miRNA HC PCR Array. The array contained miRNA-specific miScript primers, the miScript SYBR Green kit with the miScript universal primer (reverse primer), and QuantiTect SYBR Green PCR Master Mix. miRNA cDNA was quantified using a ViiA 7 Real-Time PCR System (Applied Biosystems, Waltham, MA, United States). The thermal cycling protocol was as follows: 95°C for 10 min (PCR activation step), 45 amplification cycles at 94°C for 15 s (denaturation), at 55°C for 30 s (annealing), and at 70°C for 30 s (extension), followed by fluorescence data collection. A no-template control (NTC) of RNase-free water was co-purified and profiled like the samples to measure background.

We used the 3 snoRNAs and 3 snRNAs in addition to miR-16 as endogenous normalization controls in the arrays. MicroRNA expression was quantified as delta Ct values, where Ct = threshold cycle, delta Ct = (Ct target miRNA minus the average Ct of the 7 normalization controls). Relative miRNA expression was calculated as ddCt (AD vs. CTRL) = mean dCtAD – mean dCtCTRL or ddCt (AD vs. HPC) = mean dCtAD – mean dHPC. Higher values indicate higher expression. Signals with a ddCt ≥ | 0.58|, which corresponds to a fold change of ≥| 1.5|, were considered as differentially regulated. The assay showed good technical reproducibility when repeated three times on three different days (Supplementary Figure S2).

### Quantitative PCR of Individual miRNAs

QPCR of individual miRNAs was carried out as described for the miRNA array with the following modifications: cDNA was diluted 1:100 with RNAse free water in primer specific qPCR for each miR. For qPCR validation in brains, miR-16 and SNORD95 was used as endogenous normalization controls, whereas miR-16 alone was used as normalization control for qPCR of miRNAs in neural exosomes. miR-16 has been evaluated as an endogenous normalization control in plasma, and similar plasma levels were found in AD and controls (Bhatnagar et al., 2014). Moreover, several previous studies found miR-16 levels to be consistent across AD and control brains (Hebert et al., 2008; Banzhaf-Strathmann et al., 2014). A list of all primers used for qRT-PCR is presented in Supplementary Table S1.

### Statistics

Statistical analyses were carried out using GraphPad Prism, version 8 (La Jolla, CA, United States). The Shapiro–Wilk test was used to determine normal distribution of data. Significance was determined by one-way ANOVA followed by Tukey‘s *post hoc* test for normally distributed data. Otherwise, Kruskal–Wallis test followed by Dunn‘s *post hoc* test was used. Mean age of brain and plasma donors was highly similar between diagnostic groups, and the age-distribution did not differ significantly. Thus, we did not control for age in our analyses. *P*-values are reported without correction for multiple testing because of the exploratory nature of this study. The significance threshold was set to a two-sided *p* < 0.05.

## Results

### miRNAs Are Altered in AD Brains

**FIGURE 1:**
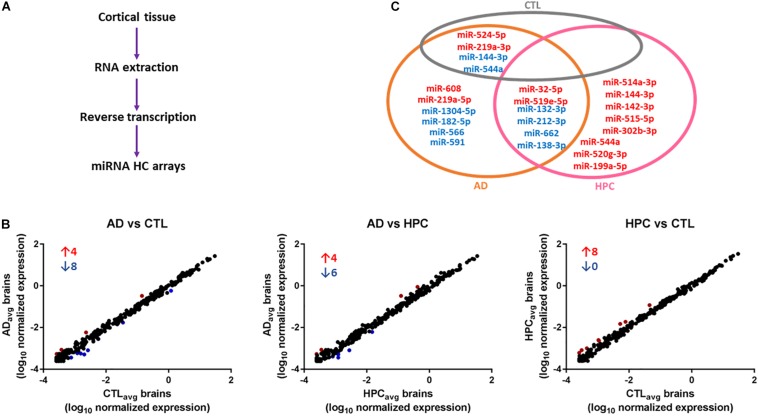
miRNAs are differentially expressed in AD compared to control and HPC brains. **(A)** Workflow of miRNA candidate discovery. RNA was isolated from cortical brain tissue of individuals who were cognitively intact (*n* = 5), AD (*n* = 5) or HPC (*n* = 5) (see Table 1 for demographic data). RNA from the 5 control, 5 AD or 5 HPC brains were pooled and used as template for cDNA synthesis. cDNA from pooled control, AD or HPC were used as templates for qRT-PCR. **(B)** Comparative analysis of profiled miRNAs between disease groups. miRNAs that are upregulated (red) and downregulated (blue) by >2-fold are indicated. Each point represents log10(2-ΔΔCt) average values from each cDNA pool run in triplicate (3 plates). **(C)** miRNAs that were significantly altered by >2-fold. A total of 16 miRNAs were dysregulated in AD brains compared to control or HPC brains. Six of those miRNAs were consistently altered specifically in AD brains (miR-608, -219a-5p, 1304-5p, -182-5p, -566, -591). Six additional miRNAs were upregulated >2-fold in AD brains compared to control, but <2-fold compared to HPC brains (miR-32-5p, -519e-5p, 132-3p, 212-3p, -662, -138-3p). Red text = upregulated miRNAs; blue text = downregulated miRNAs.

To determine whether miRNAs are dysregulated in AD brain, we pooled RNA isolated from the brains of five individuals who died with AD, five individuals who had AD pathology but were without cognitive impairment at the time of death, and five individuals who were without cognitive impairment normal and free of AD pathology (Table 1). RNA was extracted from cortical gray matter, pooled, reverse transcribed into cDNA, and cDNA was used for miRNA assays (Figure 1A). Since it is often difficult to distinguish miRNAs that are altered due to disease from normal aging, comparative analysis of profiled miRNAs was performed between AD brains relative to control, and relative to HPC. Of the 372 miRNAs that were measured, 12 miRNAs were changed >2-fold in AD brains compared to controls (4 miRNAs were up- and 8 mIRNAs were downregulated, respectively) (Table 3 and Figure 1B). Compared to HPC brains, 10 miRNAs were changed >2-fold (4 up- and 6 downregulated, respectively) (Table 3 and Figure 1B). Six miRNAs were up (miR-608, miR-219a-5p) or downregulated (miR-566, -182-5p, -591, -1304-5p), specifically in AD brains vs. both HPCs and controls (Figure 1C and Table 3). Compared to controls, 8 miRNAs were increased in HPC brains no miRNAs were decreased (Figure 1B, right panel).

**TABLE 3:** miRNAs significantly changed by ≥2-fold in AD.

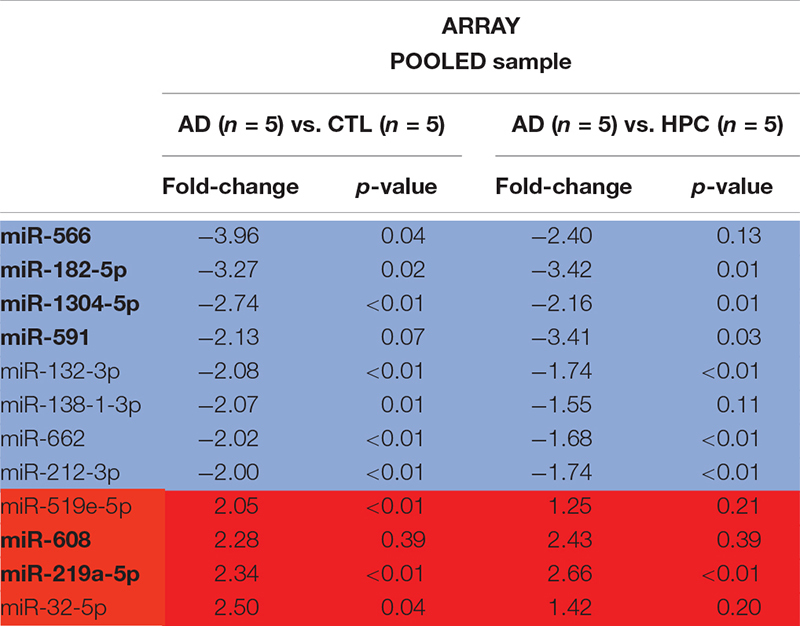

*miRs with a >2 fold change in AD compared to CTL and HPC are displayed in bold font. miRs with decreased expression in AD brain are shown blue, whereas those with a increased expression are displayed in red.*

**FIGURE 2:**
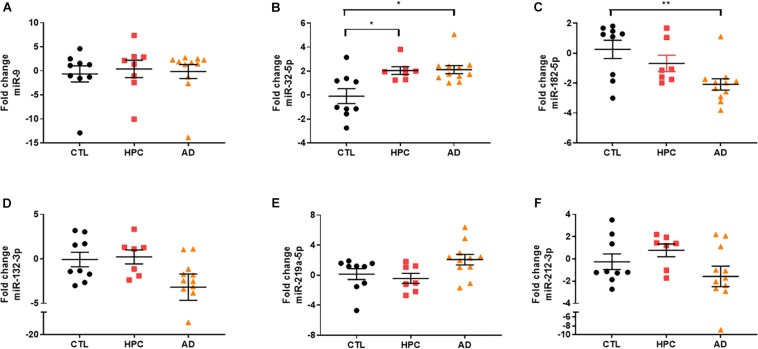
miRNA validation in AD brain tissue by qRT-PCR. miRNA candidates were measured by miRNA-specific qRT PCR. **(A)** Neuron-specific miR-9 levels, which was similar between disease groups in array analysis, were also unchanged by qRT-PCR [X^2^(2) = 0.5263, *p* = 0.769]. Two of the original hits **(B)** miR-182-5p (^∗∗^ = 0.009) and **(C)** miR-32-5p (control vs. HPC, ^∗^ = 0.049; control vs. HPC, ^∗^ = 0.025) identified by miRNA arrays were validated by qRT-PCR. **(D)** miR-132-3p was significantly changed between disease groups [*X*^2^(2) = 6.156, *p* = 0.046], but did not reach statistical significance in pair-wise comparisons. **(E)** MiR-219a-5p showed a strong trend for change between the three disease groups [*X*^2^(2) = 5.906, *p* = 0.051]. **(F)** miR-212-3p levels were not significantly changed between disease groups. Fold change was determined by 2^–ΔΔCt^ relative to miR-16. Significance was determined by Kruskal–Wallis test followed by Dunn‘s *post hoc* test.

To assess the robustness of the findings obtained using miRNA array analysis, we used miR-specific qRT-PCR to measure levels of the 12 miRNAs that were found to be most changed in our array experiments. In our initial qRT-PCR we used exactly the same pools as employed for the array analysis. The changes detected by array analysis of pooled brain RNA (Table 3) were largely confirmed by qRT-PCR analysis of individual miRs (Table 4, columns 1 and 2). Nine of 12 miRs showed the same directional and comparable magnitude changes by both arrays and qRT-PCR, but miR-1304, miR-138-1-3p, and miR-519e-5p changed in opposite directions (compare Tables 3, 4). Thereafter, we undertook qRT-PCR analysis of RNA preparations from each of the 15 individual brains used for the initial pools. When values were averaged for each disease group (i.e., for the 5 AD, the 5 HPC and the 5 controls) the direction of change detected by qRT-PCR and array analysis were in complete agreement (compare Tables 3, 4, columns 3 and 4). We then went on to use qRT-PCR to measure the levels of the same 12 miRs using RNA isolated from a total of 27 brains (11 AD, 7 HPC, and 9 cognitively intact controls). Here the results were similar to those obtained using array analysis of pooled samples (Table 3) and of the 15 individual samples (Table 4, compare columns 3 and 4 vs. columns 5 and 6). Two of these 12 miRNAs (miR-182-5p, miR-32-5p) were significantly changed from controls. miR-132-3p was significantly different between disease groups (*p* < 0.05), but did not reach statistical significance in pair-wise comparisons (control vs. AD, *p* = 0.130, HPC vs. AD, *p* = 0.094). miR-219a-5p showed a strong trend for change between disease groups (*p* = 0.051) (Figure 2). It is important to note that levels of miR-9, a neuron-specific miR, was unchanged between disease groups (*p* = 0.769). The latter finding indicates that dysregulation of miR-182-5p, miR-32-5p, and miR-132-3p are disease-specific.

**TABLE 4:** qPCR validation of miRs.

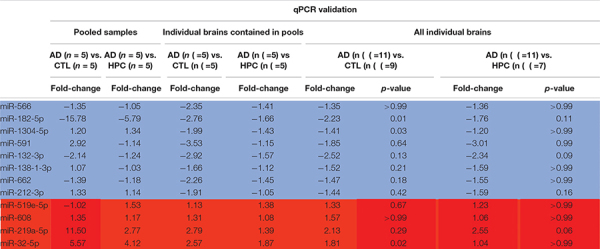

*miRs with decreased expression in AD brain are shown blue, whereas those with a increased expression are displayed in red.*

### miRNAs Can Be Detected in Neural Exosomes Derived From Blood

**FIGURE 3:**
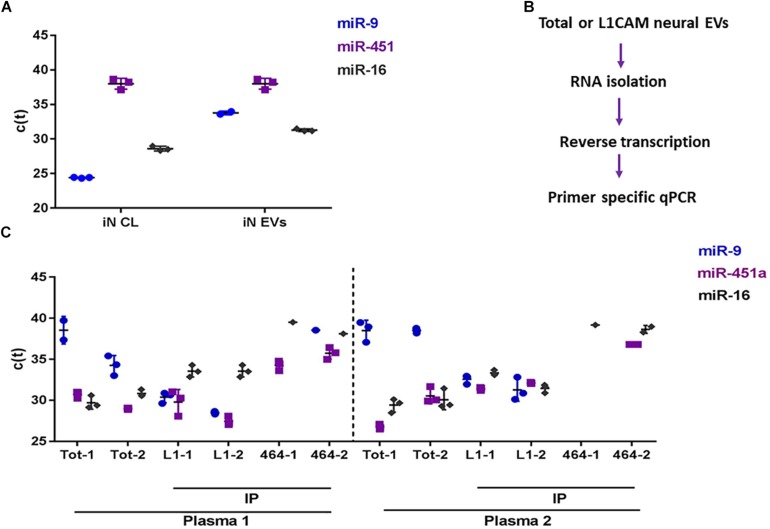
miRNA analysis of brain-derived exosomes in blood. **(A)** C(t) values for neuron-specific miR-9, plasma abundant miR-451a, and ubiquitously expressed miR-16 from induced neuronal (iNs) cell lysates (CL), and iN exosomes isolated by ultracentrifugation of iN conditioned medium. **(B)** Flow chart for the detection of miRNAs from plasma exosomes. RNAs were isolated from total plasma exosomes (Exoquick pellets), or neural exosomes immunoprecipitated (IP) from total plasma exosomes with anti-L1CAM antibody. RNA was reverse transcribed with Qiagen’s HiFlex buffer to generate cDNAs, and subsequently used as template for qPCR using miRNA-specific primers for miRNA detection. C(t) values for miR-9, miR-451a, and miR-16 from **(C)** plasma-derived total- and L1CAM (L1)- immunoprecipitated exosomes. As a negative control, an irrelevant antibody, 46-4 was used for immunoisolation. Total and IP’ed exosomes were isolated in duplicate (#1 and #2) from 2 healthy control donors (Plasma 1 and plasma 2). Low Ct values indicate high levels of miRNA, and high Ct values indicate low levels of miRNA. The reliable CT limit is ∼40.

To determine whether miRNAs can be reliably detected in neural Exosomes, we initially measured reference miRNAs in exosomes isolated from human iPSC-derived cortical neurons (iNs) (Guix et al., 2018). Ubiquitously expressed miR-16 (Chevillet et al., 2014) was detected with cycle threshold (Ct) values of 27 and 31 in iN cell lysates and their exosomes, respectively. Neuron-specific miR-9 (Coolen et al., 2013) was readily detected in both iN cell lysates (Ct = 25) and exosome (Ct = 32), whereas miR-451a, a peripherally expressed miRNA was barely detectable with ct values near the upper limit of reliable quantitation. These data are consistent with the relative enrichment of neuronal miRs in neurons and neuron-derived exosomes, and support the use of miR-9 and miR-451a as markers to assess the neuronal origin of exosomes (Figure 3A).

**FIGURE 4:**
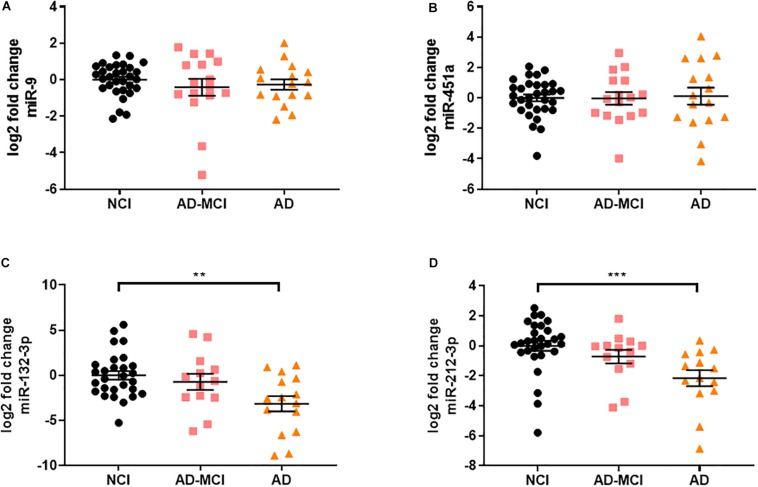
miR-132/212 is significantly decreased in AD neural exosomes isolated from plasma. **(A)** neural-specific miR-9 was abundantly detected in all measured samples and was not significantly different between disease groups [*X*^2^(2) = 1.195, *p* = 0.550]. **(B)** non-specific miR-451a were also detected in samples, but was unchanged between disease groups [F(2) = 0.035, *p* = 0.965]. Of the miRNAs significantly altered in AD brains, **(C)** miR-132 was significantly decreased in neural-derived plasma exosomes from AD subjects (^∗∗^ = 0.0333). **(D)** miR-212, a miRNA transcribed in tandem to miR-132, was also significantly decreased in AD (^∗∗∗^ = 0.001). Significance was determined by one-way ANOVA followed by Tukey‘s *post hoc* test for normally distributed data. Otherwise, Kruskal–Wallis test followed by Dunn‘s *post hoc* test was used.

Next, we examined whether we could discern a difference in the levels of miR-9 and miR451-a in total plasma exosomes vs. neural exosomes isolated from plasma using L1CAM (Figure 4B). Exosomes were isolated from the plasma of two healthy donors. RNA was extracted from total ExoQuick pellets or L1CAM-precipitated neural exosomes and used for qPCR (Figure 3B). MiR-9 was low in total exosomes (Ct = 34-37), while miR-451a was higher (Ct = 26-31). In contrast, L1CAM-isolated exosomes had high levels of miR-9 (Ct = 27-33), suggesting the L1CAM IP effectively enriched for neural exosomes (Figure 3C). The fact that we observed comparable levels of miR-451a in both total and L1CAM exosomes suggest our neural exosome preparation contained both neural and peripheral exosomes. Values for the house-keeping miR-16 were similar (Ct = 30–33) in both L1CAM and total exosomes. Notably, immunoprecipitation with the non-specific mAb, 46-4, did not lead to an enrichment of the neural miR-9. Together, these results confirm that measurement of miR-9 can be used to assess neural enrichment.

### Levels of miR-132 and miR-212 Are Decreased in Neural Exosomes in Alzheimer’s Disease

Diagnosis of AD based on clinical criteria alone is imperfect and typically 20–30% of individuals diagnosed as having AD have other neurological disorders, and considerable numbers of individuals that have AD are misdiagnosed as having other neurologic disorders (Monsell et al., 2015). To avoid use of samples with uncertain diagnose we employed samples from individuals whose clinical diagnosis was confirmed by CSF Aβ42 and tau. Using these criteria 63 subjects from the UCSD Shiley-Marcos Alzheimer’s Disease Research Center were identified (31 cognitively intact biomarker-negative controls, 16 CSF biomarker-positive MCI, and 16 CSF biomarker-positive AD) and their plasma used to isolate neural exosomes. RNA was extracted from neural exosomes, reversed transcribed and analyzed by qRT-PCR for five of the 12 miRNAs found to be altered in AD brains plus two miRNAs used to assess neuronal origin (miR-9 and miR-451a). The five miRNAs (miR-132-3p, miR-182-5p, miR-32-5p, miR-219a-5p, and miR-591) analyzed were chosen because they were consistently elevated in AD vs. control brain by >1.5-fold (Tables 3, 4). In accord with our initial experiments using L1CAM-isolated exosomes (Figure 3), neuron-specific miR-9 was readily detected in all EV preps (Ct ≤ 35) and at higher levels than the peripherally derived miR-451a (Ct ≤ 40). Similar to brain samples, fold-changes of miR-9 and miR-451a were comparable between disease groups when normalized to miR-16 (Figures 4A,B).

miRNA-219a-5p could not be reliably detected in neural exosomes, and miR-32-5p, miR-182-5p, and miR-591 were unchanged between disease groups (Supplementary Figure S3). In contrast, miR-132 was significantly decreased (Figure 4C). miR-132, a miRNA that was regulated between AD, HPC, and control brains in our study, as well as in prior studies (Wong et al., 2013; Salta et al., 2016; Pichler et al., 2017), was decreased by ∼9-fold (log2 fold decrease of 3.15) in plasma-derived neural exosomes in AD vs. controls, and ∼5.4 fold in AD vs. AD-MCI (Figure 4C). Based on this result and the fact that miRNAs within the same cluster are often co-regulated, we sought to test whether miR-212, a miRNA in tandem to miR-132, was also altered in AD. Strikingly, the levels of miR-212 were reduced 4-fold in AD samples compared to controls (Figure 4D). Interestingly, higher CSF Tau/Aβ1-42 levels showed modest, but statistically significant association with lower levels of both miR-132 and miR-212 in plasma neural exosomes. Collectively, these results suggest that concerted dysregulation of miR-132 and miR-212 occurs in AD and that measuring the levels of these miRNAs in neurally derived exosome may offer a window on changes occurring in AD brain.

**FIGURE 5:**
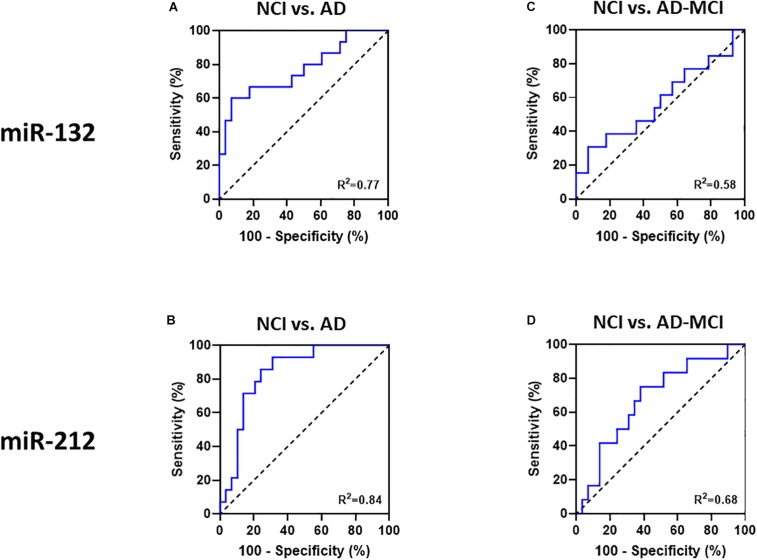
Receiver-operator-characteristics (ROC) curves demonstrate that miR-132 and miR 212 in neural exosomes evidence good separation between NCI and AD. ROC curves of **(A)** miR-132, and **(B)** miR-212 allowed good separation of NCI from AD patients. Neither **(C)** miR-132, nor **(D)** miR-212 levels in NCI vs. AD-MCI patients allowed good separation. Area under the curves (AUC) for all ROC analyses were calculated using a non-parametric approach.

Receiver operating characteristic (ROC) curves is a common tool used to assess the diagnostic utility of novel biomarkers. ROC analysis (Figures 5A,B) revealed that miR-132 levels separated controls from AD-MCI with an AUC of 0.58 (95% CI: 0.38–0.78), and controls from AD dementia with an AUC of 0.77 (95% CI: 0.61–0.93). miR-212 showed better discrimination than miR-132 between both AD-MCI and controls, and AD and controls (Figure 5). ROC analysis of miR-212 levels separated controls from AD-MCI with an AUC of 0.68 (95% CI: 0.5–0.86), and controls from AD dementia with an AUC of 0.84 (95% CI: 0.72–0.96) (Figures 5C,D). miR-212 achieves a sensitivity (the ability to predict AD cases) of 92.2% (95% CI: 68.5–99.6%), and a specificity (the ability to exclude controls) of 69.0% (95% CI: 50.8–82.7%) at the best cut-off point determined by Youden‘s J statistics. Overall, our ROC analyses indicate that measurement of miR-212 in neurally derived plasma exosomes showed sufficient diagnostic sensitivity and specificity to pursue its use as a potential screening assay for AD.

## Discussion

Despite considerable progress in our understanding of AD significant gaps remain and there is a desperate need for disease biomarkers that can offer insight about pathogenesis and which may serve as theragnostic indicators. To address this void, we exploited two exciting areas of AD research, namely miRNAs and neural exosomes. miRNAs are small regulatory molecules that post-transcriptionally repress gene expression and thereby regulate diverse biological processes, including neuronal differentiation, plasticity, survival, and regeneration (Kosik, 2006; Sambandan et al., 2017). Several neuronal miRNAs have been directly linked to AD, but there has been controversy as to which miRNAs are most important (Herrera-Espejo et al., 2019; Swarbrick et al., 2019).

In order to better define the most relevant miRNAs that are dysregulated in AD we conducted unbiased profiling of frontal cortex from three distinct groups of age-matched elderly individuals. Unlike some previous studies which compared only AD vs. controls (Salta et al., 2016; Pichler et al., 2017) we also compared AD vs. HPCs to distinguish between miRNAs that were altered by active neurodegeneration vs. those that may change in the absence of disease, but in response to amyloid and tangles. To ensure the robustness of our data we analyzed the same 5 AD, 5 HPC and 5 control samples in several different ways and then examined these 15 samples alongside additional AD, HPC and control samples. Initially, we used miRNA array analysis in an unbiased effort to profile differences between pools of RNA from AD, HPCs, and controls. Thereafter, we used miR-specific qRT-PCR to investigate the levels of 12 miRNAs that were most altered in the array experiments using both disease group pools of RNA, and RNA from individual brains. This approach yielded similar results regardless of the method used or whether RNA was from pools or individual brains, and these changes were maintained when additional brains were analyzed. Although all 12 miRNAs showed consistent fold differences (>1.5 fold) between AD, HPCs, and controls, only three miRNAs (miR-182-5p, miR-32-5p, and miR-132-3p) stood out in terms of group-based differences.

When we turned to analyze neural exosomes, we assessed miR-182-5p, miR-32-5p, and miR-132-3p, plus two other miRNAs (miR-219a-5p and miR-591) which in brain tissue showed consistent fold changes. Based on our iPSC-neuron experiments, we also used miR-9 and miR-451a to assess the neuronal origin of our exosome preparations. miR-9 is one of the most highly expressed miRNAs in the vertebrate brain (Coolen et al., 2013) and here we show that miR-9 is enriched in human iPSC-derived neurons and neuronal exosomes. As we had anticipated, the peripherally expressed miR-451a (Okamoto et al., 2018) was barely detected in human neurons or neuronal exosomes. Applying these markers to our L1CAM-isolated plasma exosomes we found that miR-9 was readily detected, whereas miR-451a was present at only low levels. It is important to note that L1CAM is not uniquely expressed in brain, but is also found in kidney cells. Therefore, we cannot completely rule out, that a small contribution may come from L1CAM-positive kidney exosomes. However, the relative enrichment of neural-to-peripheral miRNAs in L1CAM-isolated exosomes indicates that the bulk of these exosomes are of neuronal origin. Consequently, the use of miR-9 should provide the field with a to tool to differentiate between neural and non-neuronal exosomes.

Of the four miRNAs most dysregulated in AD brain only miR-132 was significantly altered in AD neural exosomes. miR-219 could not be reliably detected, whereas, miR-32 and miR-182 were reliably detected, but their levels were similar across disease groups in neural exosomes. Why these miRNAs are dysregulated in brain, but unaltered in bona fide neural exosomes is not completely clear, but it is reasonable to assume that the miRNAs measured in brain tissue are derived from many different cell types, whereas our neural exosomes are predominantly neuronal in origin. In this regard, it is interesting to note that miR-132 is one of the most abundant brain-enriched miRNAs, whereas miR-182 is highly enriched in cells of myeloid linage (Pucella et al., 2015). Indeed, in future studies it will be important to determine if the four miRNAs we found to be most changed in AD brain are dysregulated in exosomes from microglia, astrocytes or oligodendrocytes.

Notwithstanding the importance of investigating miRNAs in exosomes from other brain cell types, it is intriguing that miR-132 is downregulated in both AD brain and neural exosomes. This finding is consistent with numerous other studies which have found miR-132 to be dysregulated in AD brain and with a host of studies which tie miR-132 down-regulation to AD pathogenesis (Lau et al., 2013; Wong et al., 2013; Hernandez-Rapp et al., 2016; Salta et al., 2016; Patrick et al., 2017; Pichler et al., 2017). For instance, reductions in miR-132 appears to occur before neuronal loss and *in vitro* miR-132 protects neurons against both Aβ and glutamate (Wong et al., 2013), and overexpression of miR-132 reduces tau pathology and caspase-3-dependent apoptosis in tau transgenic mice (El Fatimy et al., 2018). Since miRNAs within the same cluster are often co-regulated, we measured miR-212 in neural exosomes. The sequence of miR-212 is closely similar to that of miR-132 and both are enriched in neurons and exert regulatory functions on neuronal survival, maturation, plasticity and memory (Wanet et al., 2012). Moreover, downregulation of the miR-132/212 cluster in the frontal cortex had been reported for patients with amnestic MCI and mild AD (Weinberg et al., 2015). Here, we found that miR-212 was strongly dysregulated in AD neural exosomes, and tended to be decreased in AD brain. Why the decrease in miR-212 was significant in neural exosomes, but not brain is unclear, but (as with miR-182) this may relate to the relative distribution of miRNAs in neurons vs. glia.

Importantly, ROC analysis indicated that measurement of miR-132-3p and miR-212-3p in neurally derived plasma exosomes showed good sensitivity and specificity to diagnose AD, but did not effectively separate individuals with AD-MCI from controls. The criteria used to identify dysregulated miRNAs in AD brain required that they were specifically altered in AD vs. both HPCs and controls. Given that at least some HPCs maybe on path to AD it is perhaps not surprising that miRNAs that are preferentially dysregulated in AD brain compared with HPC brain do not discriminate AD from AD-MCI when measured in neural exosomes. Future studies should investigate whether miRNAs that are dysregulated in HPC brain compared to control brain might discriminate AD-MCI from controls when quantified in neural exosomes. Nonetheless, quantification of miR-132 and miR-212 in neural exosomes should aid diagnosis of symptomatic patients. While this is not the population most in need of blood-based biomarkers, measurement of neural exosome miR-132 and miR-212 may have theragnostic potential and it would be particularly interesting to measure these markers longitudinally. Future studies should determine whether the observed changes in miR-132 and miR-212 can also be seen in total plasma exosomes or are specific for neural exosomes.

## Data Availability Statement

The raw data from our array experiments can be accessed through https://www.ebi.ac.uk/arrayexpress/browse.html?query= under the accession number E-MTAB-8283. All other data supporting the conclusions of this manuscript will be made available by the authors, without undue reservation, to any qualified researcher. Religious Orders Study resources can be requested at the Rush Alzheimer’s Disease Center Research Resource Sharing Hub at www.radc.rush.edu.

## Ethics Statement

The studies involving human participants were reviewed and approved by the Partners Institutional Review Board. The patients/participants provided their written informed consent to participate in this study.

## Author Contributions

DW conceived the project, designed and supervised the research, and wrote the manuscript. DC performed the exosome isolations, analyzed the brain tissue and exosomes for miR content, analyzed data, prepared figures, and wrote the manuscript. DM coded and decoded the specimen designations to ensure all experiments were done blind to the disease status of donors/patient, conducted statistical analysis, prepared figures, and wrote the manuscript. WL dissected the brain tissue and assisted with preparation of iPSC-derived neurons. DS provided the critical guidance. MM and DK provided expert guidance on the preparation of neural exosomes. DG and RR supplied archived samples and relevant clinical data. DB provided brain samples, and detailed clinical and postmortem data. All authors critically appraised the manuscript.

## Conflict of Interest

DW is an employee of Biogen Inc. The remaining authors declare that the research was conducted in the absence of any commercial or financial relationships that could be construed as a potential conflict of interest.

## Supplementary Material

FIGURE S1 Identification of AD and AD-MCI subjects based on cognitive testing and best available CSF biomarkers used for subsequent exosome isolation. A total of 63 plasma samples were obtained from the UCSD biomarker collection. CSF from these subjects was analyzed and levels of Aβ42 and tau yielded clear segregation of the disease groups.

FIGURE S2 Replicate plates within pooled disease groups show good correlation. Spearman correlation between replicates of different plates run on different days for the same sample/cDNA show high correlation with an *R*^2^ > 0.99 for all.

FIGURE S3 miR-32-5p, miR-182-5p, and miR-591 in plasma neural exosomes are unchanged between diagnostic groups. **(A)** miR-32-5p, **(B)** miR-182-5p, **(C)** miR-591, were measured in plasma neural Exosomes, but were not significantly changed (*p* > 0.05). Significance was determined by one-way ANOVA followed by Tukey‘s *post hoc* test for normally distributed data. Otherwise, Kruskal–Wallis test followed by Dunn‘s *post hoc* test was used.

TABLE S1 miRNA list and primers.
