# Supplementary figures and images for: miR-212 and miR-132 Are Downregulated in Neurally Derived Plasma Exosomes of Alzheimer’s Patients
Source: Front Neurosci. 2019 Nov 26;13:1208. doi: 10.3389/fnins.2019.01208 (PMC6902042; doi:10.3389/fnins.2019.01208)

Supplemental Figure 1

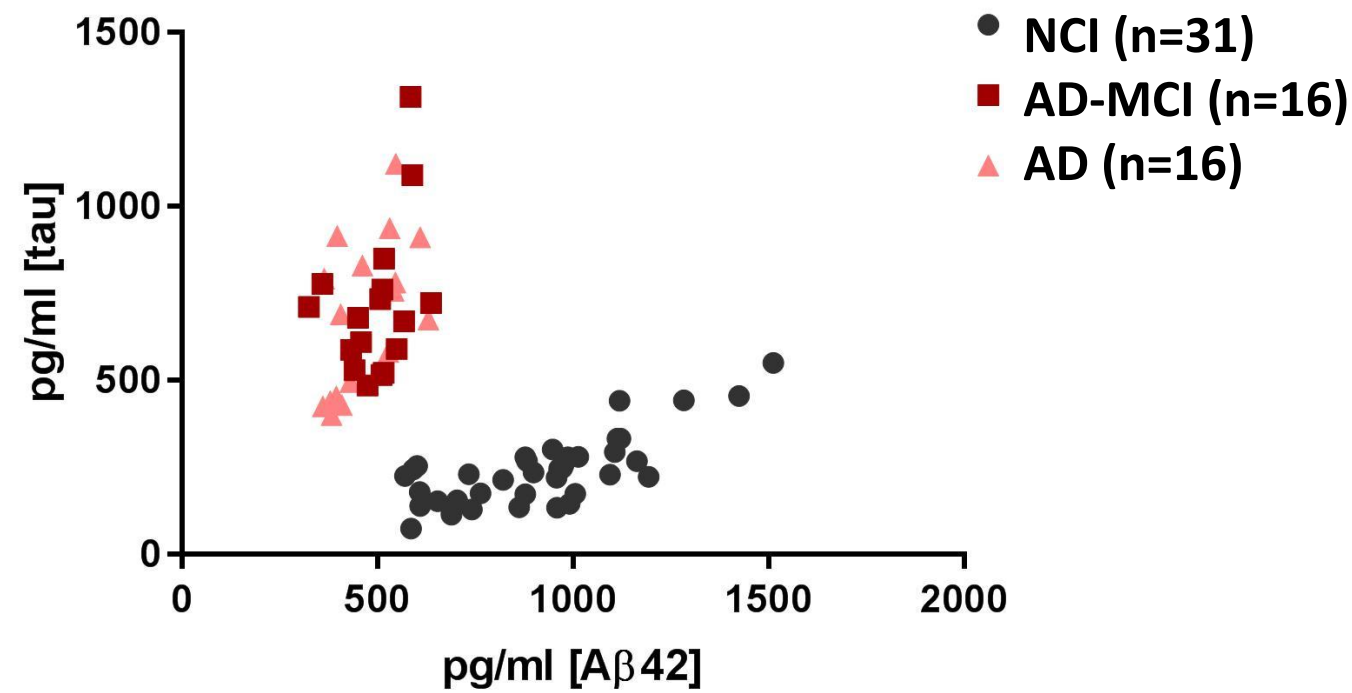

# Supplemental Figure 2

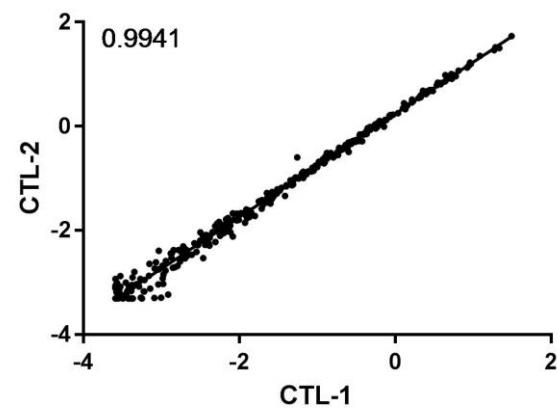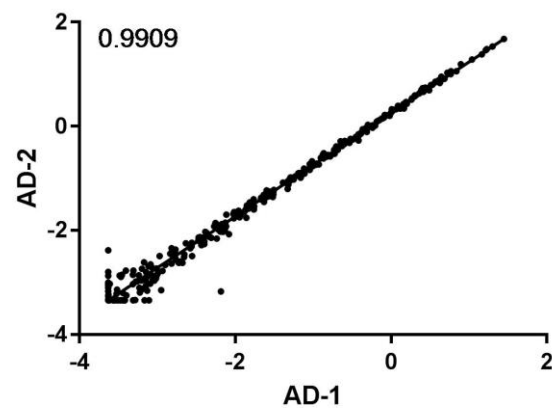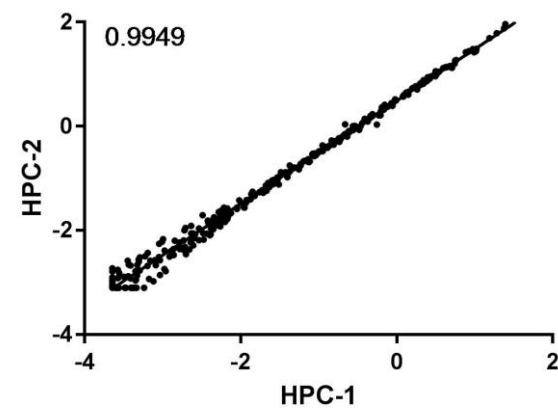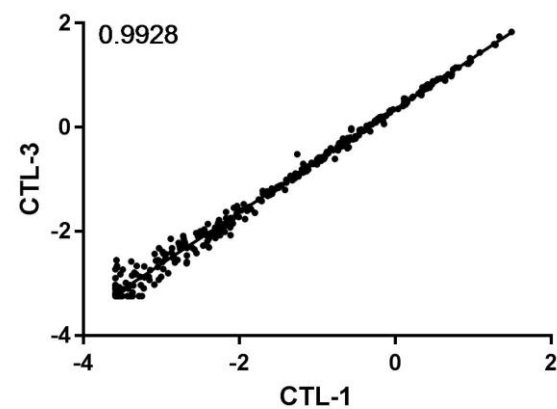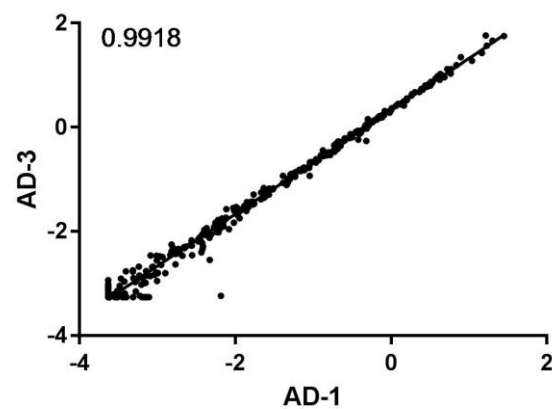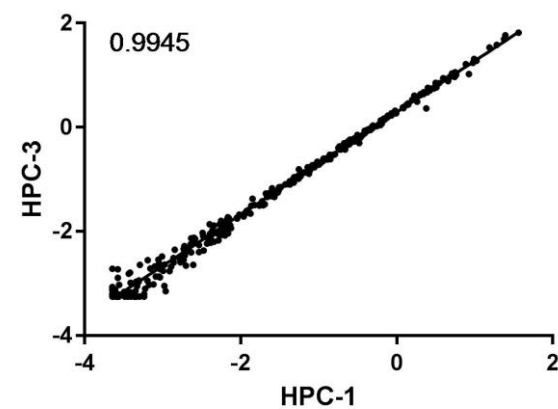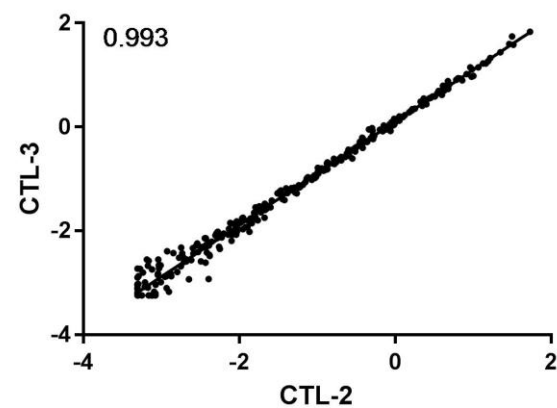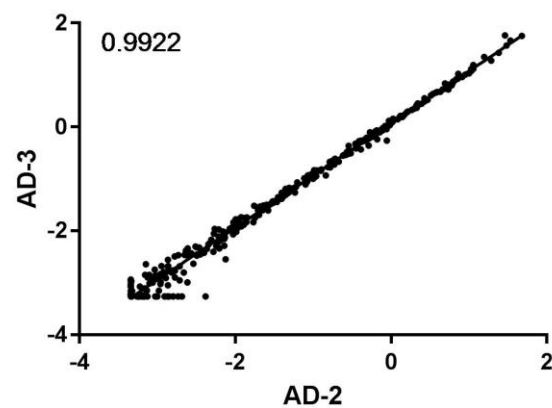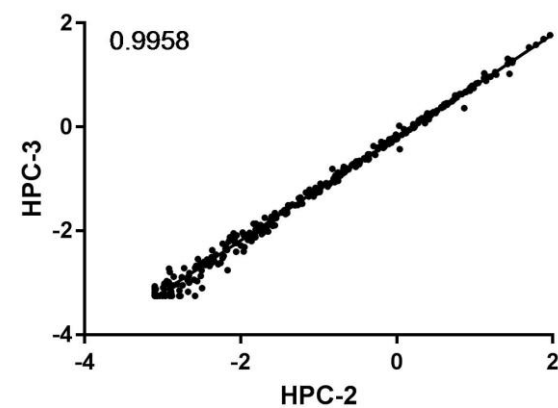

# Supplemental Figure 3

**A**

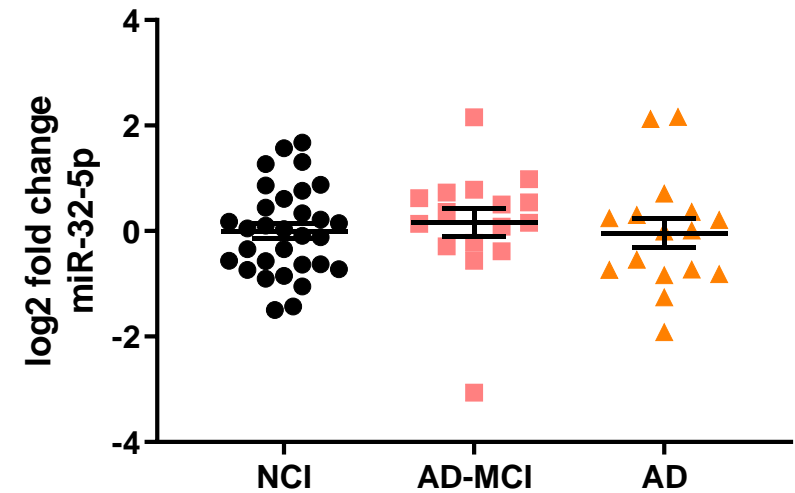

**B**

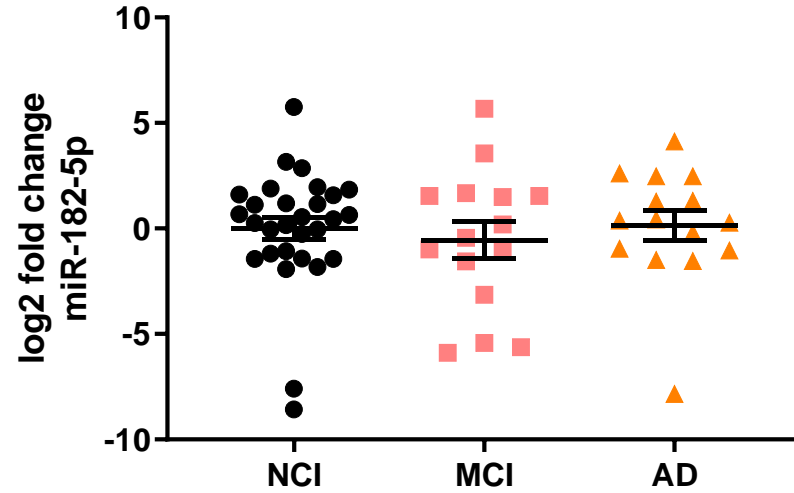

**C**

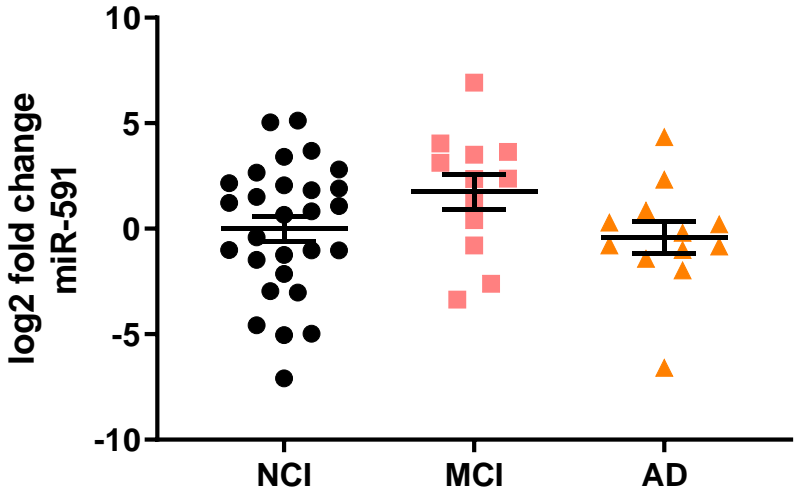

Supplement: FIGURE S1 — Identification of AD and AD-MCI subjects based on cognitive testing and best available CSF biomarkers used for subsequent exosome isolation. A total of 63 plasma samples were obtained from the UCSD biomarker collection. CSF from these subjects was analyzed and levels of Aβ42 and tau yielded clear segregation of the disease groups. [file Data_Sheet_1.PDF]
